# Fractal dimension analysis of malignant and benign endobronchial ultrasound nodes

**DOI:** 10.1186/1471-2342-14-22

**Published:** 2014-06-12

**Authors:** José Antonio Fiz, Enrique Monte-Moreno, Felipe Andreo, Santiago José Auteri, José Sanz-Santos, Pere Serra, Gloria Bonet, Eva Castellà, Juan Ruiz Manzano

**Affiliations:** 1 Pulmonology Department, Hospital Universitari Germans Trias Pujol, Planta 8, Carretera del Canyet s/n. 08916, Badalona, Spain; 2 TALP Research Center, UPC, Barcelona, Spain; 3 Pulmonology Department Hospital de rehabilitación Respiratoria María Ferrer, Buenos Aires, Argentina; 4 Pathology Department Hospital Universitari Germans Trias Pujol, Badalona, Spain; 5 Ciber de Enfermedades Respiratorias (CiBERES), Bunyola, Balearic Islands, Spain

## Abstract

**Background:**

Endobronchial ultrasonography (EBUS) has been applied as a routine procedure for the diagnostic of hiliar and mediastinal nodes. The authors assessed the relationship between the echographic appearance of mediastinal nodes, based on endobronchial ultrasound images, and the likelihood of malignancy.

**Methods:**

The images of twelve malignant and eleven benign nodes were evaluated. A previous processing method was applied to improve the quality of the images and to enhance the details. Texture and morphology parameters analyzed were: the image texture of the echographies and a fractal dimension that expressed the relationship between area and perimeter of the structures that appear in the image, and characterizes the convoluted inner structure of the hiliar and mediastinal nodes.

**Results:**

Processed images showed that relationship between log perimeter and log area of hilar nodes was lineal (i.e. perimeter vs. area follow a power law). Fractal dimension was lower in the malignant nodes compared with non-malignant nodes (1.47(0.09), 1.53(0.10) mean(SD), Mann–Whitney U test p < 0.05)).

**Conclusion:**

Fractal dimension of ultrasonographic images of mediastinal nodes obtained through endobronchial ultrasound differ in malignant nodes from non-malignant. This parameter could differentiate malignat and non-malignat mediastinic and hiliar nodes.

## Background

The ultrasound technique (US) applies sound waves (1 MHz up to 100 MHz.) that collide with tissues and thus provide energy as images. US has had a wide-ranging impact in medicine due to its low cost and by offering high resolution images.

Ultrasonography endobronchial (EBUS) has been applied as a routine procedure [1]. Three types of EBUS are currently used: EBUS radial ultra-miniature, radial and the convex or curvilinear (CP EBUS). Radial EBUS allows the evaluation of small outlying lung nodules [2]. CP USEB is the most extensively used technique because it allows to carry out mediastinal lymph node puncture (TBNA) [3,4]. Recently, several studies have demonstrated the relation between macroscopic ultrasonographic appearance and vascular patterns [5] and the likelihood of malignancy [6,7]. Although these studies have shown that some features are associated with malignancy, the evaluation of the ultrasonographic appearance depends on the observer subjectivity, and recently one study demonstrated intraobserver and interobserver disagreement [8]. Because the images contain noises mainly as a result of the reflection among adjacent surfaces, it is necessary to process them to be able to separate the real images from the noise.

The present study describes a method that improves the quality of the image, and in consequence the effectiveness of TBNA. The proposed method consists of two sections: one of having the processed image adapted to the specificities of the ultrasonography obtained by means of EBUS that eliminates the devices and specific noise of the application, and a second that characterizes the morphology of the images with the purpose of distinguishing between normal and pathological nodes.

## Material and methods

The study was developed in the Bronchoscopy Dept. of the Hospital Universitary Germans Trias i Pujol and approved by The Human Research and Ethics Committee.

EBUS-TBNA was performed in an outpatient setting using a flexible bronchoscope (BF-UC180F-OL8, Olympus Optical Co Ltd., Tokyo, Japan) with a distal probe capable of producing linear parallel scans of the mediastinal and peribronchial tissues and a working channel suited to the performance of TBNA under direct ultrasound guidance. Local anesthesia and conscious sedation were achieved using topical lidocaine spray and intravenous midazolam, respectively [BTS guidelines]. Mediastinal and lobar nodes with a short-axis diameter of ≥ 5 mm identified during the procedure were sampled under direct ultrasound visualization with a 22-gauge cytology needle specially designed for EBUS-TBNA (NA-201SX-4022, Olympus Optical Co Ltd.). The aspirates were recovered and placed on slides, fixed with 95% ethanol and stained with haematoxylin for rapid on-site evaluation by a cytopathologist. An immediate assessment was given after each pass. Nodes were classified as “normal tissue negative for malignancy” when the sample contained 40 lymphocytes per high-power field in cellular areas of the smear and/or clusters of pigmented macrophages and contained no neoplastic cells or as “metastatic” when recognizable groups of malignant cells were present. Aspirates containing only isolated dysplastic, bronchial or blood cells were considered as inadequate. In these cases the node was punctured as many times as needed to obtain adequate material.

Normal nodes were confirmed to be non-malignant by surgical procedures (patients who underwent mediastinoscopy or thoracotomy with extended nodal dissection) or by clinical and radiological follow-up for at least 18 months. In case of malignant nodes, no further confirmation was performed because the likelihood of false positive EBUS-TBNA results is very low.

### Image processing

To improve the quality, we processed the image by means of the following step sequences. The steps are standard image processing procedures that improve the quality of the image [9]. The image was first segmented to select the area of interest. A median filter was applied to remove possible spikes. Afterwards the noise of the image was reduced by a linear average 3×3 low pass filter. Local equalization with structure preserving was applied by means of a histogram 15×15. This last image will be called I1. Then I1 is filtered by means of with two orthogonal Sobel filters, which had the impulsion response (H) of:

$$H=\left[\begin{array}{c} 1\\ 0\\ -1 \end{array}\;\begin{array}{c} 2\\ 0\\ -2 \end{array}\;\begin{array}{c} 1\\ 0\\ -1 \end{array}\right]$$

which yielded images I2 and I3. The Sobel filter was used for enhancing the inner structures of the ganglia.

The final step consisted on combining linearly the filtered images I1, I2 and I3.

### Image analysis

The analysis of the images was done by means of two methods: texture and fractal dimension analysis. Texture analysis provides information of the pixels**’** intensity variability. In areas with soft texture, the range of values around the pixel is small, and when the texture is rough the range is bigger. The texture parameters to analyze are the following: a- Contrast, variance or inertia gives a measure of the intensity between a pixel and its surrounding. For a constant image the contrast is 0. b- Correlation is the relation of a pixel with its surrounding. A constant image has a correlation around 1. c- Homogeneity indicates the degree of vicinity of the elements as well as for intensity of gray. The biggest homogeneity has the value 1.

The fractal dimension of the image was computed by treating the image as a 3D object, and taking horizontal slices of it at different intensity levels. Therefore for each intensity level we created a binary image, where we assigned the value white to the intersection of the surface with the slice and to the inner pixels. In other words, for each gray level we created and image and assigned the white value to the set of pixels with that gray level and the pixels inside the regions. The black value was assigned to the other pixels. The result was that for low values of gray level most of the figure was white, and as the gray level increased, the images begun to take shapes like fiords, as the gray level continued to increase, islands appear, and finally, the whole image finally is black. The algorithm computed the inner area (white space) and its perimeter. We assumed a perimeter model of the node inner structure as follows: Log (Perimeter[n]) = k + α Log(Area[n]). Parameters k and α were computed after a least squares linear regression was applied. The fractal dimension is the α value that models the increase of the perimeter as the area of the figure increases.

A possible characterization of the structures in the images could be done by means of the box counting dimension [10]. We decided not to use it in this problem due to various difficulties.

a) The box counting dimension assumes a binary image with two different zones. The box counting method consists of computing the fractal dimension by counting the boxes that overlap the border between regions at different scales (sizes) of the boxes. This assumes that there is a specific threshold that characterizes the different areas of interest, and the gray level information of the image is lost. In our case, as the different structures of the tissue are reflected in the intensity (or gray level) of the image, we computed the regression of the log perimeter vs log area, not on the different scales of the boxes, but on the variation of the log area/log perimeter relationship at different gray scale levels.

b) The second difficulty was that the size of the areas of interest in the ultrasonography was small (of the order of less than 100x100, depending on the selected area) and therefore the estimate of the fractal dimension by means of the box counting method would have been unreliable, due to the lack of points.

Statistical analysis of differences in image parameters between independent groups were performed with a Mann_Whitney U test. In addition, a receiver-operator characteristic (ROC) curve was applied to measure the capacity of the method to discriminate between neoplasic and non neoplasic nodes.

The Image processing part of the study was made using the Matlab programming language. The texture parameters were computed by means of the subroutines of the same name (i.e. Contrast, variance or inertia ) in the 'Image processing Toolbox', and the fractal dimension part was programmed in Matlab. Statistic analysis was developed with Statistica v.12 (StatSoft, Inc 2013. Tulsa.USA).

## Results

**Figure 1:**
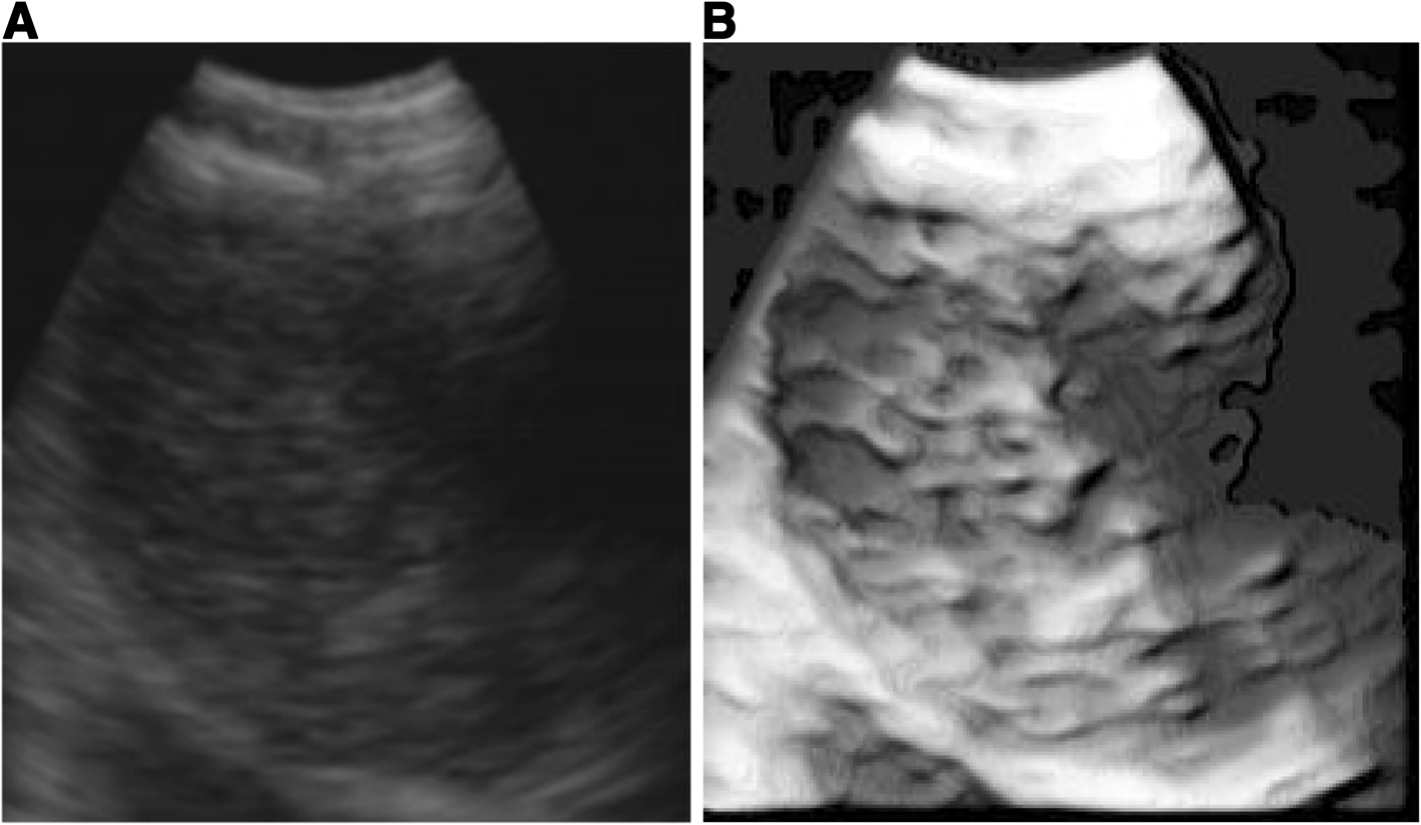
**Non processed EBUS image (1-A) and processed image (1-B).** Image details are emphasized in image **B** with respect to image **A**.

Table 1 shows the histological results of 23 biopsied mediastinal nodes. Twelve nodes were malignant.The ultrasound images were processed in order to improve the quality of EBUS image and to enhance the details, as can be seen in Figure 1 (1-A non processed image, 1-B processed image).

**Table 1:** Characteristics and results of lymph nodes

**ID**	**Type**	**Cytological diagnosis**	**Type**	**Station**	**Size (mm)**
1	Malignant	Carcinoma	Breast C	7	23.8
2	“	Carcinoma	Breast C	7	28.9
3	“	Squamous		4 L	8.9
4	“	Squamous		11 L	19.1
5	“	Adenocarcinoma	NSCLC	7	13.2
6	“	Squamous	NSCLC	4 L	6.7
7	“	Adenocarcinoma	NSCLC	4R	9.1
8	“	Squamous	NSCLC	4 L	17.1
9	“	SCLC		4R	11.2
10	“	Adenocarcinoma	NSCLC	4R	13.4
11	“	Adenocarcinoma	NSCLC	7	23.0
12	“	Adenocarcinoma	NSCLC	4 L	9.2
13	Benign	Normal		4R	8.2
14	“	Normal		4 L	4.1
15	“	Normal		7	11.8
16	“	Normal		7	14.2
17	“	Normal		4R	5.9
18	“	Normal		4R	8.3
19	“	Normal		4R	8.6
20	“	Normal		11 L	10.6
21	“	Normal		7	10.4
22	“	Normal		4 L	9.8
23	“	Normal		4R	9.1

Histological characteristics of 23 needled lymph nodes.

NSCLC: non-small cell lung cancer.

SCLC: small cell lung cancer.

Breast C: breast cancer.

Table 2 shows morphologic parameters and fractal dimension of 23 biopsied lymph nodes. Processed images showed that fractal dimension was lower in neoplasic with respect to non neoplasic nodes. There were no differences between both groups in the morphological parameters.

**Table 2:** Morphological image parameters

	**Processed image**		
	**Neoplasic**	**Non Neoplasic**	**All**
Fractal dimension	1.47(0.09)	1.53(0.10)*	1.50(0.10)
Contrast	0.35(0.09)	0.40(0.14)	0.37(0.12)
Correlation	0.95(0.01)	0.95(0.02)	0.96(0.01)
Homogeneity	0.87(0.03)	0.86(0.04)	0.65(0.03)

Mann–Whitney U test for independent groups.

*P < 0.05. Significant difference between neoplasic and non neoplasic nodes.

All values are in mean (SD).

Morphological parameters and fractal dimension of 23 biopsied lymph nodes. The table shows that fractal dimension was lower in neoplasic with respect to non neoplasic nodes. There were no differences in morphology parameters between neoplasic and non neoplasic images.

**Figure 2:**
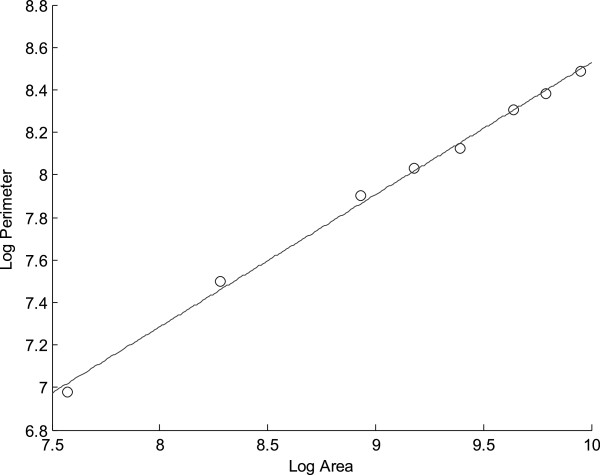
**Relationship between the log area and the log perimeter of a node image.** The slope of the straight line would give us the form in that the log- perimeter grows linearly with the log-area. In this example the relationship agrees with a lineal model. *Log*(*Perimeter*[*n*]) = *K* + *αLog*(*Area*[*n*]). Coefficients: α = 0.62, K = 2.31.

**Figure 3:**
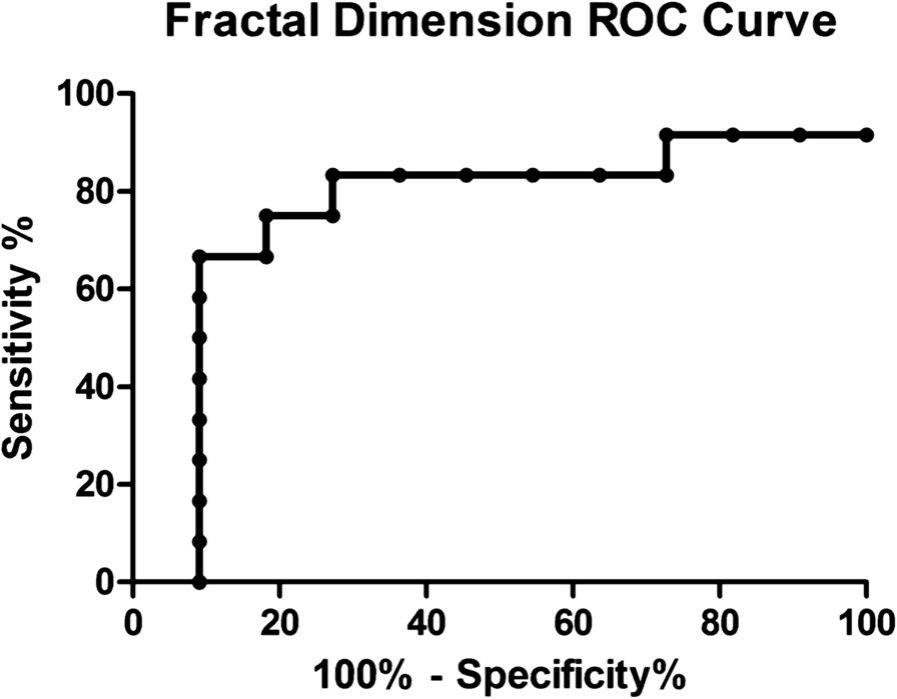
**ROC curve of the fractal dimension parameter.** The area under the curve was 0.76 (p < 0.03).

Figure 2 shows the relationship between the log area and the log perimeter. The slope of the straight line would give us the form in that the log-perimeter grows linearly with the log-area. In this example the relationship agrees with a lineal model. Except for fractal dimension, there were no differences in morphological parameters between images (Table 2). On the other hand, the fractal dimension was smaller in malignant lymph nodes (Mann–Whitney U test for independent groups, p < 0.05).Figure 3 shows the ROC curve of fractal dimension parameter. The area under the curve quantifies the overall ability of the fractal dimension measure to discriminate between neoplasic and non neoplasic nodes. The area under the curve was 0.76 with Std Error of 0.11 (p < 0.03).

## Discussion

In this work we studied the relationship between parameters that describe the texture and fractal dimension of endobronchial ultrasonographic images of mediastinal nodes and the likehood for malignancy. In both raw images as well as enhanced ones it was found that there is a statistical difference between malignant and non-malignant nodes in terms of fractal dimension.

The introduction of EBUS-TBNA has provided a significant advance in the staging and diagnosis of lung cancer and other malignancies in a safe and minimally invasive procedure [11]. The analysis of the ultrasonographic appearance of the nodes has been applied to predict malignancy. Fujiwara et al. studied morphologic characteristics of lymph nodes by means of a multivariable analysis that included round shape, distinct margin, heterogeneous echogenicity and presence of coagulation necrosis [6]. The authors found that these morphologic characteristics are independent predictive factors for predicting malignancy. Echogenicity was the parameter with the most validated punctuation. The authors did not apply the automatic process of the image, but only qualitative subjective evaluation. Nguyen et al. applied for the first time the second order grayscale texture feature analysis in EBUS [12]. In their study, 52 malignant nodes and 48 benign ones were analyzed. They found that malignant nodes have a higher difference in first and second order texture parameters in relation with benign nodes, using as distinctive features in texture parameters based on first and second order statistics. It should be noted that images were not pre-processed in order to maintain the same real time quality image. On the other hand, the differences in textures after enhancing the image were not significant. This can be attributed to the fact that the processing smoothed the image, eliminated spurious peaks, and enhanced the inner structures of the nodules. This processing that improved the visual appearance of the details of the nodes, changed the texture of the image.

An interesting aspect of the proposal in this paper of introducing the fractal index α, is that this index is complementary with respect to the texture parameters. This complementariness arises from the fact that the fractal index is adapted to the shape of the internal structures of the nodule, and therefore appears as significant after the enhancement of the image. On the other hand the raw image has too much noise, which gives rise to artifacts when computing the fractal index. The fractal dimension is a real number that generalizes the concept of ordinary dimension for geometric objects. This process also provides data regarding phenomena like deformation, remodeling, breakup and repair. Cancer in general is associated with a disruption of tissue architecture due to the interaction between cells and stroma [13], and fractal-shape parameters could be descriptors of the cell-stroma system. On the other hand, there is a fractal relationship between the degree of apparent heterogeneity of local tissue and the resolution of the measurement, when heterogeneity provides no uniformity in the cell organs examined.

Fractal dimension has been applied in ultrasound echo signals to detect tissue tumors [14,15]. Texture parameters and fractal Higuchi dimension of the ultrasound series detected prostate cancer in small tissue regions with an accuracy of 91% [15]. Zheng et al. [16] applied fractal Brownian motion and k means cluster analysis to detect breast cancer with a recognition rate of 94.5% for malignant tumors. In the present work, we analyzed 23 nodes (12 of them malignant), and applied an algorithm to compute the inner area (white space) and its perimeter. We assume a power model between the perimeter of the inner structure of the ganglia and the area. Difference of fractal dimension between malignant and non malignant nodes was significant, and less in malignant nodes. A possible cause of this slight reduction in fractal dimension of malignant nodes is that cell membranes spread to take the form of a lower energy structure like a circle, therefore, diminishing the fractal dimension of a neoplasic node [13]. In this way, Kikuchi et al. [17] showed that sonography of solid components in cystic epithelial ovarian cancers had a fractal structure, and the mean fractal dimension decreased from 1.26 for serous intracystic components to 1.18 for clear cell adenocarcinoma. In our study the mean fractal dimension was more than 1, meaning the topological line dimension, and it decreased from 1.53 for benign nodes to 1.47 for malignant nodes, the same proportion of the Kikuchi study.

We believe that the principal limitation of our study is the relatively small number of analyzed nodes, but the objective was to describe the fractal nature of the ultrasonographic images of mediastinal nodes. A future application and validation of the present technique could be developed to distinguish between malignant nodes and other non-malignant pathologies that affect mediastinal nodes (such as tuberculosis and chronic inflammatory diseases like sarcoidosis). We should always try to obtain pathological reference diagnosis from suspicious lymph nodes, but in the future, image analysis could assist the bronchoscopist regarding the likelihood to malignancy of the node, as well as the most suspicious region of the node to sample. In consequence, we believe that fractal dimension can constitute a new EBUS parameter to take into account. To our knowledge, this is the first study that applies fractal dimension analysis to EBUS images.

## Conclusion

Fractal dimension of ultrasonographic images of mediastinal nodes obtained through endobronchial ultrasound differ in malignant nodes from non-malignant. This parameter could assist the bronchoscopist to differentiate malignant and non-malignant mediastinic and hiliar nodes.

## Competing interest

The authors declare that they have no competing interest.

## Authors’ contributions

JAF: Participated in the design of study, statistical analysis and manuscript writing. EM: Participated in the study design, statistical analysis and manuscript writing, as well as software programming that computed the fractal dimension of the data and the image processing part. FA: Participated in the study design, manuscript writing and bronchoscopy explorations. SJ: Participated in bronchoscopy explorations and contributed in design and manuscript writing. JS: Participated in bronchoscopy explorations and contributed in design and in the writing and revising of the manuscript. PS: Participated in bronchoscopy explorations and contributed in design and revision of the manuscript. EC: Participated in bronchoscopy explorations and in the design and revising of the manuscript. JR: Participated in the design of study, statistical analysis and manuscript writing. All authors read and approved the final manuscript.

## Pre-publication history

The pre-publication history for this paper can be accessed here:

http://www.biomedcentral.com/1471-2342/14/22/prepub
